# Three dimensions of speech coherence in people with early psychosis and their family members

**DOI:** 10.1038/s41537-025-00703-0

**Published:** 2025-12-17

**Authors:** Derya Çokal, Abdulrahman Aloraini, Claudio Flores Palominos, Cemal Demirlek, Burcu Verim, Berna Yalınçetin, Emre Bora, Wolfram Hinzen

**Affiliations:** 1 Institute for German Language and Literature I Linguistics, University of Cologne, Cologne, Germany; 2 Department of Information Technology, College of Computer, Qassim University, Buraydah, Saudi Arabia; 3 Department of Translation and Language Sciences, Universitat Pompeu Fabra, Barcelona, Spain; 4 Department of Psychiatry, McLean Hospital, Harvard Medical School, Belmont, MA USA; 5 Department of Neurosciences, Health Sciences Institute, Dokuz Eylul University, Izmir, Turkey; 6 Department of Psychiatry, Dokuz Eylul University, Izmir, Turkey; 7 Department of Psychiatry, Melbourne Neuropsychiatry Centre, University of Melbourne and Melbourne Health, Carlton South, 3053 VIC Australia; 8 ICREA (Institució Catalana de Recerca i Estudis Avançats), Barcelona, Spain

**Keywords:** Biomarkers, Human behaviour

## Abstract

Fundamental to coherence in discourse is referential structure – identifying entities through noun phrases (NPs, e.g. *a man*; *that large cat*) and tracking them in discourse. But coherence is also mediated by conceptual-semantic structure, as tracked through semantic similarity relations between words, and by predictability (“perplexity”). Alterations in speech coherence have long been noted in schizophrenia spectrum disorders (SSD) along all three of these dimensions. These are likely connected, but have largely been studied in isolation. This study targeted them together, in Turkish-speaking people with a first episode of psychosis (FEP, *n* = 53), youths at ultra-high risk (UHR, *n* = 64) of SSD, people with a family history of psychosis (FHP, *N* = 39), and 34 neurotypical controls (NC). In FEP, we confirmed a pattern previously attested in chronic SSD, of fewer definite NPs (e.g., *this bald man*), more “bare” NPs—i.e., lacking functional elements such as *this/a* —, and an unexpected random distribution of indefinite NPs; moreover, referential anomalies (unclarities of reference) were more prevalent in all groups relative to NC. FEP and UHR also showed higher word-to-word semantic similarity, and FEP larger image-to-text bimodal semantic distance, mirroring a pattern previously attested in English. NP-related variables related to both semantic similarity and perplexity, with crucially different correlational patterns seen for definite vs. indefinite NPs. Together, these results substantiate crosslinguistic evidence of a referential disturbance in early psychosis, partially extending to the extended schizophrenia phenotype, while additionally supporting that referential structure is closely integrated with conceptual semantics and the probabilistic structure of speech.

## Introduction

It is impossible to use language without generating *referential meaning*: words that we retrieve from memory for speech, need to match objects that we talk about, and the situations that we find ourselves in. Referential meaning is anchored in our knowledge of the *conceptual meaning* of words as encoded in semantic memory, e.g., *elephant* or *fly*. These concepts entail general information about the world, such as that elephants are typically tall and do not fly. Beyond such information as encoded in word meaning, all human languages have devices that systematically regulate reference to *specific* objects and events in the world, e.g., *This elephant flew*, where we restrict our general concept of elephants to a particular context and convey information about it. In English, these devices involve functional structure in grammar, e.g., determiners such as *a*, *the*, *that*, or *those*, which introduce noun phrases (NP, e.g., *tall elephant*), or pronouns (e.g., *it*), which can track references across discourse. Each of these devices regulates reference in different ways, e.g., *the* allows anaphoric reference to an elephant mentioned before, as in *The elephant that flew*. This happens in interaction with the grammatical configuration of the sentence, so as to give rise to a spectrum of forms of reference that range from the most generic (e.g., *I don’t like elephants*) to *indefinite* (*I saw an elephant*) to definite and pronominal (e.g.*, The elephant / it was happy*) forms^1,2^. In this way, the determiner system along with sentential grammar orchestrate an interplay of different NP types that jointly create referential coherence over narrative time or discourse. This claim holds crosslinguistically, insofar as we classify NP subtypes by grammatical function (definite/indefinite; anaphoric/specific/generic) rather than morphologically through the presence of articles such as *the/a*, which are missing in languages like Turkish. There, definiteness/specificity is conveyed through other grammatical means, such as differential object marking (accusative case marker -(y)I on specific/definite objects), demonstratives (*bu/şu this/that*), the numeral *bir* (one) functioning as an indefiniteness marker, and the discourse context (see Table 1 for examples).

**Table 1:** Noun phrase (NPs) types with examples and explanations.

	NP types		
DPs	Indefinite DPs	Yakışıklı bir adam.lit. handsome a man. (‘a handsome man’)	The NP (‘man’) is used with an indefinite determiner *bir* (‘a’), which introduces a new referent into the discourse.
	Definite DPs	Bu adam çalışan olmalı. lit. this man worker be must. (‘This man should be a worker.’)	The NP adam ‘man’ is used with a demonstrative determiner ‘bu/this’, which points/refers to a specific entity in the picture.
Bare NPs	Anaphoric	Adam tarlada çalışıyor lit. Man land-in work-3^rd^-ing. (‘A man is working in the field.’)	The NP adam (‘man’) is used without a determiner (bare) but refers to an NP mentioned in the previous discourse.
	Specific due to case-marking	Adam tarlayı sürüyor. lit. Man land-the. plow-3rd-ing. (‘A man is plowing the land.’)	The NP tarla (‘land’) is used with the accusative case marking –(y)I, which signals that the person is talking about a specific entity.
	Indefinite	Ev-ler var. Lit. house-s there-are. (‘There are houses.’)	The plural morpheme *-ler* on the bare NP ev (‘house’) evler semantically is an indefinite quantification over a set of houses.
	Generic	Köy hayatını anlatıyor. (‘It is about the village life.’)	The NP refers generically to a village lifestyle.
	Bare-residual	Kız var. lit. girl there is. (‘There is girl.’)	The NP kız (‘girl’) is not used with any determiners, case markers or morphemes, does not refer to any previously mentioned entity, and is not specifically marked for indefiniteness.

*DPs* Determiner Phrases (NPs introduced by a determiner).

*Bare NPs* NPs occurring without determiners.

Referential structure plays a crucial role in coherence, which has been widely noted to be affected in schizophrenia spectrum disorders (SSD)^3–5^, though its specific linguistic dimensions remain unclear. Referential disturbances have often been noted to occur in SSD^6,7^, and anomalies of language are of diagnostic and prognostic importance in SSD^8^. Previous studies have specifically identified shifts in the relative proportions of different types of NPs in SSD, more pronounced in, but not restricted to those patients with formal thought disorder (FTD), in English^9^, Turkish^10^, and Mandarin Chinese^11^. Recent work also shows these anomalies to be detectable automatically using large language models (LLMs)^12^. Alterations affect not merely the proportions of NP types, but also their distribution over narrative time, specifically how random this distribution is^10^. Moreover, referential anomalies—use of NPs such that referents cannot be tracked by a listener – are over-represented in *definite* NP types^9,13^. An unresolved question concerns sensitivity of these alterations to disease stage and genetic liability. Most existing studies have examined chronic patients. Targeting the early end of the schizophrenia spectrum by recruiting patients after a first episode of psychosis (FEP) or at ultra high risk of psychosis (UHR), can address possible chronicity and medication effects. In turn, parallel language anomalies of family members of affected individuals can uncover genetic biases, as have previously been documented^14,15^, and target trait-like vulnerabilities across the spectrum, partially independent of symptom severity or illness stage.

With the arrival of large language models (LLMs), which can perform language tasks at human levels, the field of language studies in SSD has increasingly shifted towards automated analyses that use embedding techniques. These encode words through high-dimensional vectors (“embeddings”) that capture the co-occurrence patterns of words in large corpora, viewed as a proxy of their meaning^16^. The cosines of the angles between such vectors are commonly interpreted as capturing their semantic similarity^17^. This in turn connects to the idea that such distances can capture a decrease in coherence in SSD through a decrease in mean cosine similarity between word pairs, alluding to the concept of “loosening of associations”. While a number of studies have empirically demonstrated a decrease in mean semantic similarity using earlier language models^18–20^, several recent studies across a number of languages have found increased semantic similarity between words in patients with SSD as compared to healthy individuals, conceptualized as indicating a “shrunk” semantic space^21–23^. These contradictory findings block the interpretation of semantic similarity as measuring coherence and raise several further issues, specifically the dependence of the semantic similarity effect on the language model (e.g., fastText vs. transformer models, which diverge in^21^), and whether the shrinkage effect is severity- and stage-of-illness related. Another issue is how semantic similarity is related to referential structure, and whether the former notion can illuminate the latter, regardless of the LLM used, given unclarity over whether LLMs capture referential meaning at all^24^. It is clear that meaning and grammatical-level reference always work together in an integrated fashion in language, as all reference in language is ultimately based on the lexical concepts we understand and retrieve from semantic memory in the form of words, and all words are referentially used. Linking common semantic similarity metrics from LLMs to variables relating to referentiality at a grammatical level, is therefore an important current desideratum.

The same applies to the relation between referentiality and common metrics extractable from LLM embeddings, such as the information-theoretic metric of “perplexity” (mean surprise) of a word given its context, which has been found to be elevated in SSD^23^ and to relate to brain-functional changes in this disorder^25^. Next-word predictions of LLMs are computed from embeddings and depend on grammatical structure fixing reference in connected speech.

### Present study

The present study aimed to profile grammatically different NP types that are distinguished by their referential function, and to relate these referential variables to semantic similarity and perplexity metrics as extracted from LLMs, in the same data. This was to empirically connect three dimensions of meaning previously studied largely in isolation from one another. We did this in Turkish, a non-Indo-European language, where previous evidence^10^ has shown shifts in the distributions of NP types in a chronic schizophrenia group, using a categorization of NP types first established in English^9^ (an Indo-European language). Across these language families, as well as Sinitic (Mandarin^11^), a comparable pattern of referential anomalies with chronic patients has now been observed, despite differences arising from language-specific pronoun and article systems across these languages. Our overarching hypothesis was that the constructs under study—referential NP structure, semantic similarity, and predictability—tap into universal aspects of language organization. We also hypothesized that they would be sensitive to SSD at either a stage- or trait-level, and that they would associate with each other.

To explore this, we targeted groups that are early on the psychosis spectrum—individuals after a first episode of psychosis (FEP) and individuals at ultra high-risk of psychosis (UHR)—as well as the extended schizophrenia phenotype, i.e., family members of individuals with SSD (FHP). Based on the previous study of Turkish^10^, we predicted that bare NPs (lacking determiners) would increase in prevalence in SSD while definite NPs (featuring determiners) would decrease. In turn, for semantic similarity, based on previous evidence in English^23^, we also predicted, specifically for the FEP and UHR groups, an increase in word-to-word cosine similarity, a decrease in how semantically similar descriptions of pictures are to these pictures themselves, and an increase in perplexity of next-word predictions as calculated from transformer-based contextual LLMs^26^. The FHP group was added for exploratory purposes to investigate trait-related genetic liability, and we predicted that more subtle language changes would be seen in this group, overlapping with those in the other groups. Regarding relations between NP distributions and semantic variables, our general prediction was that changes in the distribution of NP types—representing shifts in referential strategies in connected discourse—would predict semantic similarity. This prediction was theoretically grounded: as noted, conceptual-semantic similarity and referentiality are not independent dimensions in the organization of language. Specifically, when an indefinite determiner is used, e.g., *a woman*, the reference is established mainly via the lexical concept involved: the referent is identified through the property of being a woman. By contrast, *the woman* would shift the focus to the referent being identical to one previously mentioned, while *her* would lose all lexical-descriptive content altogether. Definite reference, therefore, could allow to establish coherence partially independently of the lexical concepts retrieved, while indefinites (and generic NPs, as in *Elephants don’t fly*) depend on these to a greater extent. We therefore predicted that definites and indefinites would predict semantic similarity in potentially opposite directions, with definites likely diminishing word-to-word semantic similarity and indefinites increasing it. In turn, when computing sentence-to-picture (“bimodal”) semantic similarity in picture-description tasks, more definites would predict closer sentence-to-picture alignment (increased bimodal semantic similarity), while indefinites would do the opposite. As mishandling reference is expected to lead to lesser coherence, we also predicted that NP variables would predict increased levels of perplexity in discourse as measured with LLMs.

## Results

### Referentiality

As a first step, we calculated incidence rates of different types of NPs. Examples and definitions are provided in

**Fig. 1:**
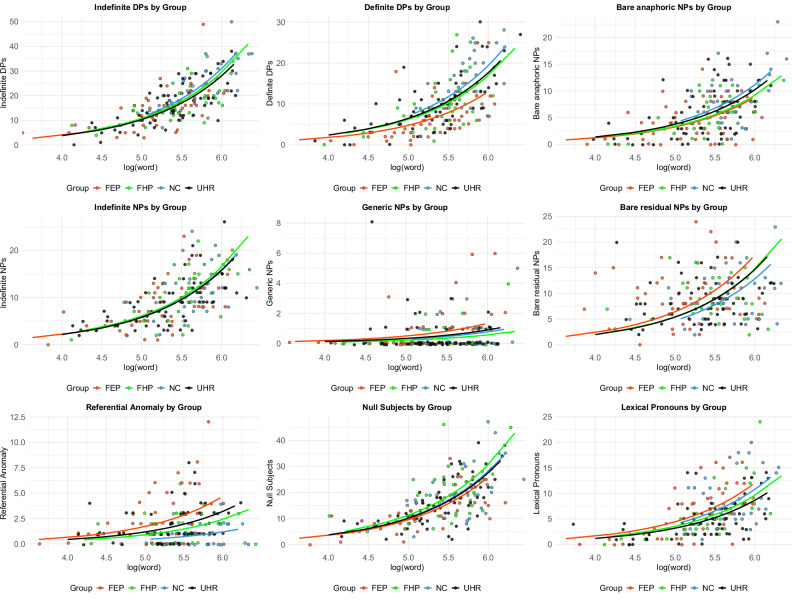
Predicted rates of linguistic variables by log (word) and group. Bare specific NPs were not plotted due to insufficient instances.

For descriptive statistics comprising means and standard deviations, see Table 2. Results from Poisson regression models are presented in Table 3, where differences in the incidence (log) rates for the linguistic variables in the groups relative to neurotypical controls are indicated. As shown, the FEP group exhibited a significant decrease in the rate for NPs introduced by a functional item (determiner) indicating definiteness (identified as “Determiner Phrases” (DPs) below). The same applied to bare anaphoric NPs (see Tables 1 and 2, Fig. 1). This pattern reversed in a grammatically simpler type of “bare” NP (lacking functional structure, anaphoricity, and definiteness), here called bare-residual NPs, with the FEP group showing an increase in incidence rates relative to controls. The UHR group showed a significant decrease in lexical pronouns. However, given the modest effect size and the marginal nature of this finding, we note this result is exploratory and in need of replication. The three FEP, UHR, and FHP groups all broadly followed the same pattern involving an increase in referential anomalies relative controls (Table 3). These results confirm a previous result in a chronic sample, that the referential structure of discourse is altered in psychosis, affecting specifically definite-anaphoric NPs and referential anomalies^10^.

**Table 2:** Means and standard deviations of referentiality variables across groups.

Variables	NC *M* (s.d.)	FEP *M* (s.d*.*)	UHR *M* (s.d.)	FHP *M* (s.d.)
Number of utterances	49.94 (13.86)	40.77 (12.69)	41.55 (15.82)	46.00 (14.90)
Number of words	312 (92.92)	211.34 (87.88)	229.84 (94.29)	252.41 (92.48)
Indefinite DP	23.97 (9.07)	15.34 (8.01)	15.92 (8.44)	18.44 (8.00)
Definite DP	15.00 (6.63)	6.81 (5.60)	10.09 (6.72)	10.44 (6.80)
Bare anaphoric NP	8.65 (4.84)	4.64 (3.51)	5.81 (4.71)	5.74 (3.50)
Bare specific NP	0.06 (0.24)	0.00	0.20 (0.54)	0.03 (0.16)
Bare indefinite NP	12.29 (5.12)	8.32 (4.96)	8.98 (5.04)	10.21 (5.18)
Bare generic NP	0.59 (1.16)	0.70 (1.37)	0.52 (1.20)	0.33 (0.81)
Bare-residual	9.94 (5.06)	9.17 (4.98)	8.39 (4.18)	9.18 (4.02)
Null subject	21.79 (10.01)	13.58 (7.41)	15.75 (9.07)	19.28 (9.49)
Lexical pronoun	8.35 (4.73)	6.32 (5.17)	4.95 (3.83)	6.05 (4.75)
Referential anomalies	0.91 (1.00)	2.45 (2.48)	1.81 (1.78)	1.46 (1.35)

*NC* neurotypical controls, *FEP* first-episode psychosis, *UHR* ultra-high risk, *FHP* family history of psychosis, *NP* noun phrase, *DP* determiner phrase.

**Table 3:** Incidence rates (log scale/) of different NP types in the groups relative to neurotypical controls (reference).

Variables & Group	*B*	*Se*	*z*	*p*	*q*
Indefinite DP					
(Intercept)	−2.57	0.04	−73.26	0.00	0.00*
FEP	−0.06	0.05	−1.15	0.25	0.32
UHR	−0.10	0.05	−2.20	0.03	0.06*
FHP	−0.05	0.05	−0.99	0.32	0.32
Definite DP					
(Intercept)	−3.02	0.14	−21.08	0.00	0.00*
FEP	−0.37	0.07	−5.20	0.00	0.00*
UHR	−0.00	0.07	−0.03	0.98	0.98
FHP	−0.10	0.07	−1.39	0.16	0.20
Education	0.02	0.01	1.52	0.13	0.19
Age	−0.01	0.01	−1.65	0.10	0.19
Bare anaphoric NP					
(Intercept)	−3.55	0.19	−19.18	0.00	0.00*
FEP	−0.22	0.09	−2.41	0.02	0.05*
FHP	−0.15	0.09	−1.60	0.11	0.13
UHR	−0.08	0.09	−0.87	0.38	0.38
Education	0.03	0.01	1.81	0.07	0.11
Age	−0.02	0.01	−1.86	0.06	0.11
Bare specific NP					
(Intercept)	−6.84	1.79	−3.81	0.00	0.00*
FEP	−16.96	2006	−0.01	0.99	0.99
UHR	1.40	0.83	1.69	0.09	0.28
FHP	−0.41	1.25	−0.33	0.74	0.89
Education	0.06	0.17	0.36	0.72	0.89
Age	−0.12	0.11	−1.14	0.25	0.51
Bare indefinite NP					
(Intercept)	−3.14	0.15	−21.33	0.00	0.00*
FEP	−0.00	0.07	−0.05	0.96	0.96
UHR	0.01	0.07	0.20	0.84	0.96
FHP	0.06	0.07	0.82	0.41	0.61
Education	0.01	0.01	0.84	0.40	0.61
Age	−0.01	0.01	−1.42	0.16	0.47
Bare generic NP					
(Intercept)	−6.24	0.60	−10.38	0.00	0.00*
FEP	0.51	0.28	1.78	0.07	0.22
UHR	0.13	0.31	0.41	0.68	0.87
FHP	−0.40	0.37	−1.07	0.29	0.57
Education	0.01	0.04	0.23	0.82	0.87
Age	−0.01	0.03	−0.17	0.87	0.87
Bare−residuals					
(Intercept)	−3.43	0.06	−61.81	0.00	0.00*
FEP	0.30	0.07	4.16	0.00	0.00*
UHR	0.08	0.07	1.12	0.26	0.26
FHP	0.12	0.08	1.57	0.12	0.15
Null subject					
(Intercept)	−2.64	0.04	−70.60	0.00	0.00*
FEP	−0.10	0.05	−1.97	0.05	0.10
UHR	0.01	0.05	0.10	0.92	0.92
FHP	0.07	0.05	1.28	0.20	0.27
Lexical Pronoun					
(Intercept)	−3.67	0.18	−20.07	0.00	0.00
FEP	0.10	0.08	1.21	0.23	0.44
UHR	−0.26	0.09	−2.85	0.00	0.01*
FHP	−0.10	0.09	−1.05	0.29	0.44
Education	−0.01	0.01	−0.67	0.50	0.50
Age	0.01	0.01	0.84	0.40	0.48
Referential anomalies					
(Intercept)	−5.47	0.37	−14.99	0.00	0.00*
FEP	1.26	0.20	6.18	0.00	0.00*
UHR	0.87	0.21	4.09	0.00	0.00*
FHP	0.63	0.23	2.73	0.01	0.01*
Education	−0.02	0.02	−0.99	0.32	0.39
Age	0.00	0.02	0.11	0.91	0.91

*FEP* first-episode psychosis; *UHR* ultra-high risk; *FHP* family history of psychosis.

*Highlights the significant values after FDR corrections (*q*).

### Distances and distributions of NP types over narrative time

**Fig. 2:**
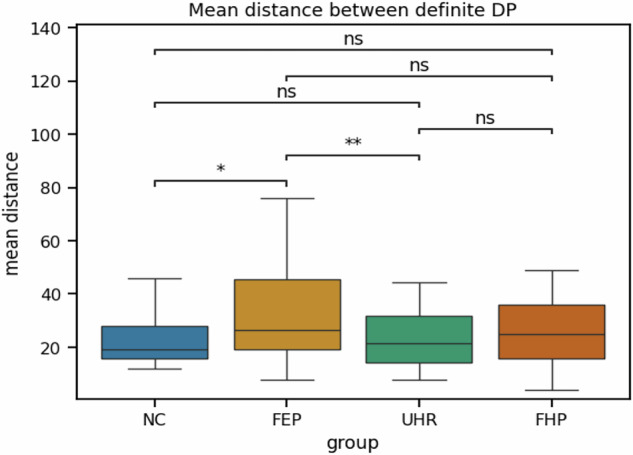
Between-group comparisons of the average mean distance of definite DPs. Neurotypical Controls (NC), First-episode Psychosis (FEP), Ultra-High Risk (UHR), and Family History of Psychosis.

As a second step, we calculated the average distances between, and distributions of, occurrences of four macro-types of referential expressions (definite DP, indefinite DP, bare indefinite NP, and bare-residual). Mean distance is the average number of tokens between successive occurrences of the same macro-types within a narration (Note: Larger values indicate sparser recurrence). Using this metric, definite DPs were more sparsely distributed in FEP than in NC: There was an increase in the mean distance of definite DPs in FEP relative to NC, as expected from the lower incidence rates of these DPs (FEP: *M* = 33.57, SD (20.97); NC: *M* = 26.43, SD (19.53), *p* = 0.018); as well as relative to UHR (*M* = 23.98, SD (11.57), *p* = 0.006 (see Fig. 2).

For the same four NP macro types, exponential distributions of their instances over narrative time are not expected, as a narrative would typically begin with indefinites introducing entities, and definites later picking them up. Definites and indefinites are therefore not expected to show a random arrival process, or a “memory-less” pattern. In line with this expectation, the Poisson null hypothesis (i.e., exponential inter-arrival times) could be rejected for indefinite DPs, in NC, FHP and UHR (NC: *p* = 0.013; FHP: *p* = 0.004; UHR: *p* = 0.031). By contrast, the same null hypothesis (*p* = 0.192) failed to be rejected for indefinite DPs in FEP, which means that previous indefinite DPs do not change the chance of the next one. Put differently, in FEP, the production of this type of NP can be modeled in a memoryless fashion, such that previous indefinite DPs do not influence the probability of future events.

### Semantic similarities and perplexity across groups

**Fig. 3:**
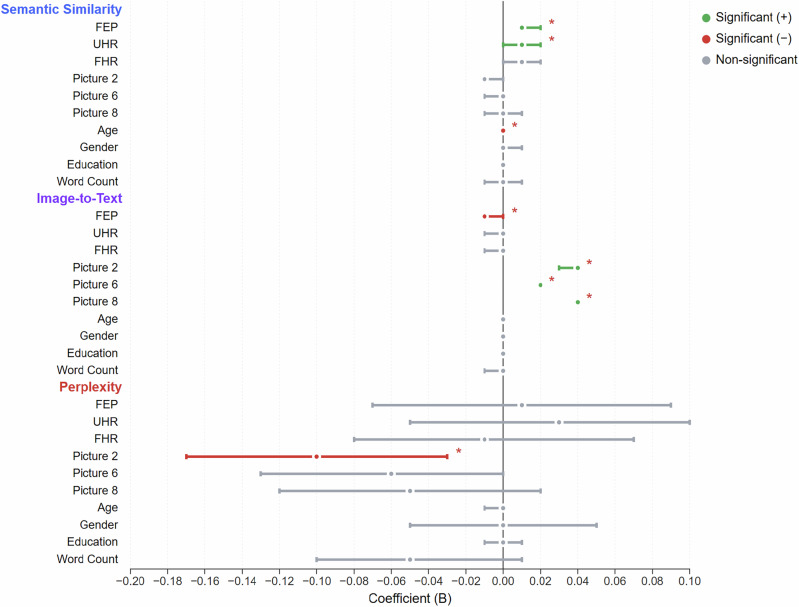
Forest plot showing regression coefficients with 95% confidence intervals for group effects (FEP, UHR, FHR) and covariants on three outcome measures. *Green* indicates significant positive effects, *red* indicates significant negative effects, and *gray* indicates non-significant effects (*q* < 0.05 after correction). First-episode Psychosis (FEP), Ultra-High Risk (UHR), and Family History of Psychosis (FHP).

Word-to-word semantic similarity was operationalized as the mean cosine between adjacent token embeddings from a monolingual Turkish BERT. Embeddings from a transformer-based LLM for Turkish (a monolingual BERT model^26,27^) were retrieved for every token and consecutive semantic similarities were computed pairwise from them. FEP and UHR both showed higher semantic similarity compared to controls (FEP: *B* = 0.013, *q* = 0.008; UHR: *B* = 0.012, *q* = 0.021), and FEP also showed a decreased image-to-text (“img2txt”) semantic similarity, when using the bimodal Jina-CLIPv2 model, which embeds the image and the description of it separately and then computes the semantic similarity between them (FEP*: B* = −0.006, *q* = 0.006; see Fig. 3; see Table S1 for semantic similarity and perplexity across groups).

### Relationships between NP types and (unimodal and bimodal) semantic similarities

**Fig. 4:**
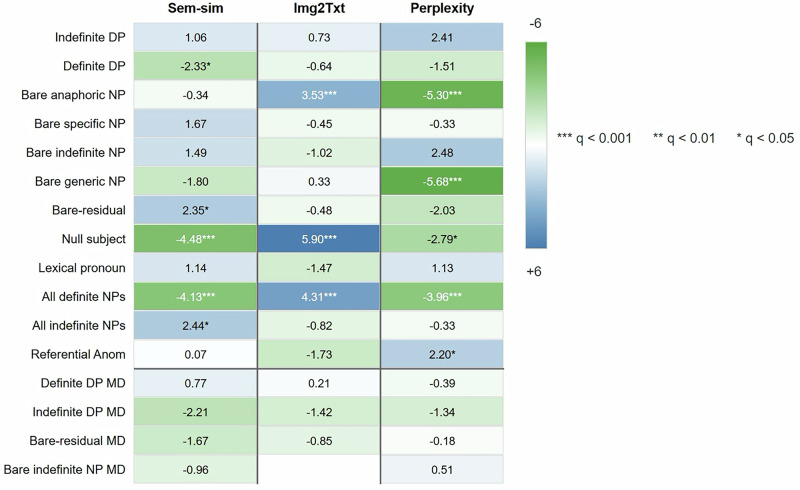
Associations of NP types and NP mean distances with word-to-word and image-to-text semantic similarity, and perplexity. Displayed numbers represent z-scores. The larger the z score, the denser the color. Determiner Phrase (DP), Noun Phrases (NPs), Anomalies (Anom) and Mean Distance (MD).

Image-to-text (“img2txt”) similarity was computed as the cosine between image and caption embeddings using Jina-CLIPv2. Definite DPs (*B* = −0.10, q = 0.04), null-subjects (*B* = −0.15, *q* = 0.00), and all definite NPs (*B* = −0.09, *q* = 0.00) were negatively associated with word-to-word semantic similarity scores. In contrast, all indefinite NPs (*B* = 0.09, *q* = 0.03) and bare-residual NPs (*B* = 0.10, *q* = 0.04) showed positive associations (see Fig. 4 and Table S2 for results of a Generalized Estimating Equations (GEE) model controlling for group, age, gender, education, and the specific picture described; and Table S3 and Table S4 for predictions within particular groups). In addition, definite NPs, specifically bare-anaphoric NPs, null subjects, and all definite NPs, were significantly positively related to bimodal semantic similarity between the narration and the image (Bare-anaphoric NPs: *B* = 0.09, Null subject: *B* = 0.09, All definite: 0.05, all *p* < 0.001) (Fig. 4; see Table S5 for deviance goodness-of-fit test results for the GEE models).

### Relationships between NP variables and perplexity

*Perplexity* was defined as the mean negative log-probability of tokens under a Turkish Transformer language model (lower values indicate higher predictability). Bare anaphoric NPs (*B* = –2.48, *q* = 0.00), bare generic NPs (*B* = –3.96, *q* = 0.00), null subjects (*B* = –0.83, *q* = 0.03), and all definite NPs (*B* = –0.82, *q* = 0.01) were negatively associated with perplexity, indicating greater predictability of the text from the viewpoint of the LLM as the incidence rates of these NPs increased (See Fig. 4; Table S2 and Table S6). On the other hand, indefinite DPs (*B* = 0.84, *p* = 0.02, *q* = 0.06) and bare indefinite NPs (*B* = 1.37, *p* = 0.01, *q* = 0.07) showed a trend-level *positive* relation with perplexity, after correction (Fig. 4, Table S2 and Table S6 for predictions within particular groups). Referential anomalies were positively correlated with perplexity (*B* = 3.11, *p* = 0.03, *q* = 0.03). No significant group differences in perplexity were found across FEP, FHP, and UHR, even before correction.

### Relationships between linguistic variables and symptoms (Brief Negative Symptom Scale (BNSS), Scale for the Assessment of Positive Symptoms (SAPS) Negatives, & SAPS Formal Thought Disorder)

Spearman’s partial correlations were computed between clinical symptom scales (BNSS, negatives and FTD) and linguistic measures, including all definite NPs vs. all indefinite NPs, all definite mean distance vs. all indefinite mean distance, and NLP metrics (similarity, image-to-text alignment, and perplexity). Across all clinical scales, there were no statistically significant relationships. Partial correlations controlling for group, age, education, and gender revealed no significant associations between linguistic variables and clinical symptoms (all *p* > 0.05 after FDR correction) (see Table S7 for the partial correlation between clinical symptoms of FEP, FHP, and UHR and linguistic variables).

## Discussion

This study sought to connect three dimensions of the meaning of linguistic expressions in spontaneous speech, which have so far been largely studied separately from each other in psychosis: the referential meaning of words, by which they relate to the world, their distributional meaning as captured with embeddings from LLMs, and the predictability of words in the contexts in which they occur. Using an annotation scheme for NP types previously used in a chronic SSD group in the same language^10^, we firstly replicated the pattern seen there in chronic schizophrenia in FEP: a reduction in the proportion of definite and anaphoric NPs, along with an increase in a class of bare NPs, which lack anaphoricity and definiteness. Given that the previous study^10^ focused on patients with and without formal thought disorder (FTD), like previous similar studies in English^9,13^, this pattern of a reduction in definiteness is thus neither restricted to chronic patients nor to those with FTD. Moreover, indefinite NPs, which are not expected to show a random pattern of appearance during a narrative, showed such a profile in the FEP group, consistent with previous findings in chronic SSD in Spanish^28^, in Turkish^10^, and in Mandarin Chinese^11^.

Consequently, there is now considerable evidence that referential strategies as encoded through the NP system in human languages show divergences from early one in the disease history of psychosis. The UHR group, on the other hand, did not show the above patterns, though they (and they alone) were linguistically anomalous in underproducing lexical pronouns. Given the marginal effect size, we regard this result as exploratory and emphasize the need for replication in independent samples. Pronoun production is a well-documented anomaly in psychosis^29^, though this does not apply to a pattern of overproduction of third-person pronouns specifically. In Turkish, lexical pronouns tend to have a contrastive or emphatic referential function, as opposed to the anaphoric uses of null pronouns (marked only in morphology). These typological features make direct cross-linguistic comparisons of pronoun use less straightforward. Consequently, part of the pattern we observed may reflect Turkish-specific properties rather than psychosis-specific effects. However, other findings—such as reductions in definite and anaphoric NPs—have been reported across Indo-European^9^ and non-Indo-European languages^10,11^, suggesting that certain referential anomalies in psychosis generalize across language families. Future cross-linguistic work will be essential to disentangle universal patterns of referential disruption in psychosis from language-specific realizations.

Regarding distributional meaning, we partially replicated a pattern that was specific to a high-symptomatic FEP and a clinical high-risk group in a previous English study^23^, of higher word-to-word semantic similarity jointly with lower bimodal text-to-image semantic similarity, though in our study, this pattern again did not extend to UHR. Contrary to the findings in refs. ^23^, and the general expectation that speech in psychosis is harder to predict, the information-theoretic measure of perplexity did not distinguish any groups in our study. A possible reason could be the higher symptom load in the (largely unmedicated) FEP group in the previous study^23^. While FHP did not show any divergence in NP type variables, they aligned with FEP and UHR in showing an increase in referential anomalies relative to controls, though the effect was reduced compared to that in these other groups. This alignment might indicate a more profound and generalizable linguistic change related to a genetic liability for psychosis, indicating a partially shared vulnerability, which as such may not correlate with clinical symptoms.

Reference, as mediated by grammar and word meaning are crucially distinct dimensions of semantics, yet necessarily integrated with each other in normal language use, as noted above. Key to our study therefore was the relationship between them, and between them and perplexity, a metric derived from embeddings. A striking pattern appeared in the relation between referential NP-based variables and LLM-based distributional semantic variables, in line with our theoretically grounded prediction: There was a negative association with word-to-word semantic similarity centered on *definite* types of NP (i.e., definites), while there was a positive association with *indefinite* types of NP (i.e., indefinites): as incidence rates of definite NPs increased, semantic distances between words increased, while they decreased with indefinites. What had motivated our prediction was that indefinites, lacking referential specificity, ground meaning in concepts, not in reference, allowing a speaker to stay within a lexical-conceptual space. When this space is transgressed, on the other hand, and entities have been established that are now independent of the concepts with which they have been identified, coherence can be built partially independently of the concepts involved, and a tendency towards a shrinking semantic space (narrower semantic distances between concepts) can be overcome. The flipside of this would be that when more definite NPs are used, the image described can be better targeted, leading to higher text-to-image semantic similarity, as indeed we found; while with more independent, picture descriptions may be too guided by internally anchored conceptual associations, leading to lower text-to-image semantic similarity.

Although perplexity did not distinguish our groups, referential NP variables showed a relation to perplexity as well. Specifically, perplexity increased with more referential anomalies, and with lesser use of definite-anaphoric NPs, which were reduced in FEP, while again, indefinites showed an opposite (positive) relationship, with more perplexity seen with more indefinites produced. Breaking this pattern, bare generic NPs, which are at the opposite (indefinite) end of the referential spectrum compared to definites, showed the same negative relationship as definites. It is not clear why this latter pattern occurred. The important message here is that the probabilistic structure of speech is highly integrated with its grammatical structure, specifically its NP structure. This is expected on theoretical grounds, as the predictability of words in context, as computed by LLMs, changes with grammatical structure in that context, to which LLMs are sensitive. The flow of words becomes more predictable precisely as grammar is imposed on it.

Notably, no relations to clinical symptoms were found with any of our linguistic variables, whether referential, semantic similarity related, or information theoretic. This is surprising particularly for FTD, to which referential structure has previously been shown to be sensitive^10^. Relations between semantic similarity-related metrics and symptoms, on the other hand, have shown high variability in the past^22,30,31^.

One likely contributing factor for the absence of correlations in our study is the relative homogeneity of symptom profiles within groups. Correlation analyses require sufficient variability, but within our group-based design, clinical ratings tended to cluster in narrow ranges (e.g., low scores in HC and FHP, moderate–high scores in FEP, two low scores and one heterogeneous score in UHR), which restricts the possibility of detecting possible associations. In addition, our FEP participants were clinically stabilized (30% reduction in positive symptoms, HDRS < 12), restricting symptom variance and potentially masking state-dependent relationships. We also note that clinical rating scales are relatively coarse measures and may not capture the specific linguistic mechanisms reflected in our linguistic metrics. In addition, our sample size may have limited statistical power to detect correlations, and the cross-sectional design precludes testing whether associations emerge more strongly when symptoms fluctuate over time. Future work with broader variability in symptom severity—either through larger samples or longitudinal designs—will be necessary to evaluate the diagnostic and prognostic value of these linguistic measures.

At the same time, the absence of symptom correlations does not preclude clinical relevance. Our findings show that linguistic anomalies were detectable at the group level in FEP, UHR, and FHP despite the lack of associations with symptom ratings, suggesting that they may reflect trait-like or liability-related features of psychosis rather than transient state markers. This perspective highlights a potential utility distinct from traditional diagnostic scaling: language-based measures may provide insight into underlying disease mechanisms that shape coherence and reference independently of overt symptoms. In the longer term, such markers could contribute to early detection strategies and inform intervention targets by identifying vulnerabilities that are stable across disease stages, and by linking them to neural mechanisms of coherence processing where symptom correlations may be absent. While this could question the diagnostic utility of linguistic NLP-based metrics, there also is evidence that such metrics can relate to brain signal when symptom scores do not^25^, underlining their promise for advancing the understanding of disease processes. We note that linguistic coherence measures may serve as trait markers for psychosis vulnerability rather than state markers of symptom severity. This positions them as potentially valuable for risk identification rather than symptom monitoring.

## Limitations

Several limitations of the present study should be acknowledged. First, the sample sizes were modest, particularly for the NC (*n* = 34) and FHP (*n* = 39) groups. At the same time, our sample sizes are larger or comparable to those in previous work^9,15,32–34^ on language production in individuals with schizophrenia using computational, linguistic, or probabilistic model, and our inclusion of three distinct groups (FEP, UHR, FHP) provides a window onto different stages and liabilities of psychosis. We therefore view our findings as an important first step that motivates future large-scale investigations.

Second, while our study design allowed us to connect referential NP variables with LLM-based measures of semantic similarity and perplexity, the cross-sectional nature of the data limits conclusions about developmental trajectories or predictive value for illness progression. Longitudinal designs will be crucial to test whether the observed linguistic patterns serve as state markers, trait markers, or predictors of clinical outcomes.

Finally, although we used state-of-the-art LLMs to derive semantic and probabilistic measures, these tools remain black-box models whose interpretability is limited. Our results should therefore be understood as pointing to robust associations rather than mechanistic explanations. Future work combining NLP with neuroimaging and experimental paradigms may help to clarify the mechanisms by which referential and semantic processes jointly contribute to coherence in psychosis.

## Conclusions

NLP-based research in psychosis has created a large variable space, the basic dimensions of which need to be clarified. This study has shown that three such dimensions—referential structure as encoded in NPs, semantic similarity as encoded from LLM embeddings, and predictability of speech—are affected in psychosis, although not uniformly across groups or measures, and that they relate to each other in systematic ways. A specific split, documented in previous studies and languages, has again transpired here between definite and indefinite types of NPs, which make different predictions for semantic similarity patterns or how words are predictable. These patterns are clearly attested in early psychosis following remissions after a first episode, while they largely did not extend to an even earlier group at ultra-high risk.

## Methods

**Fig. 5:**
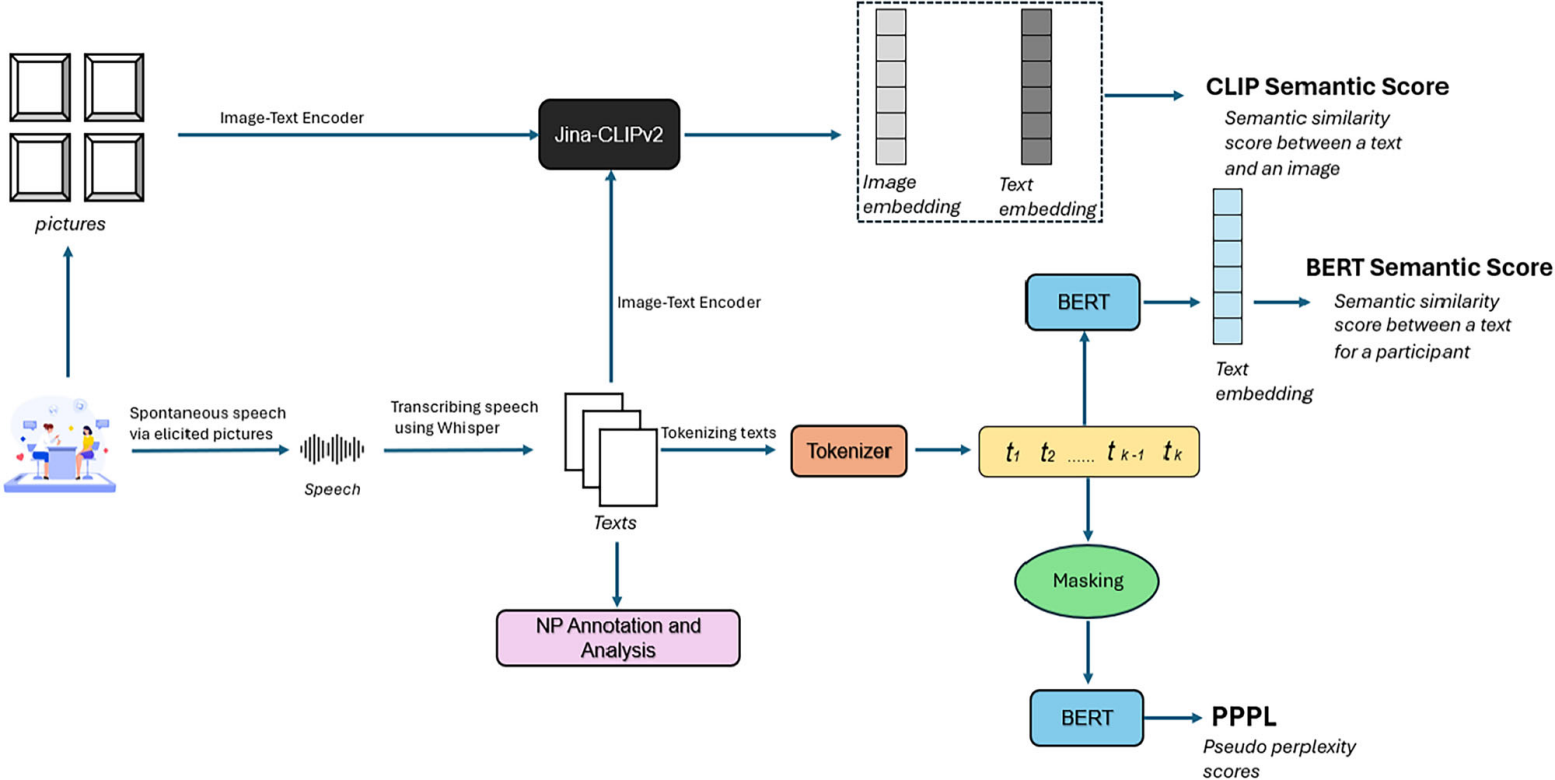
Schematic of the workflow of the study. Workflow for computing multimodal coherence metrics from picture-elicited speech. Participants’ spontaneous speech is elicited through pictures and transcribed with Whisper. Texts are tokenized and used for NP annotation and further linguistic analysis. Image–text embeddings are generated with Jina-CLIP to derive CLIP-based semantic similarity between pictures and spoken descriptions. Text embeddings from BERT are used to compute semantic similarity scores and pseudo-perplexity (PPPL) via masked language modeling.

Figure 5 illustrates the workflow of the study.

### Participants

The study sample consisted of 53 participants with first-episode psychosis (FEP), 64 ultra-high-risk individuals (UHR), 39 individuals with a family history of psychosis (FHP), and 34 neurotypical controls (NC). In 80% of the FHP sample, individuals had a first-degree relative with a schizophrenia diagnosis; the rest had multiple second-degree relatives. Patients with FEP were enrolled through the first-episode program of the Early Intervention in Psychiatric Disorders Unit (ETAP) at the Department of Psychiatry, Dokuz Eylul University Hospital, Izmir. FE participants with a schizophrenia spectrum disorder who had a history of a first psychotic episode within the last 12 months and were all currently clinically stable. The criteria for clinical stability were to include individuals not having acute psychotic or affective episodes and those who had not changed their medication in the last 4 weeks due to symptom exacerbation. Additionally, a reduction of over 30% in total positive symptom scores was required, along with a Hamilton Depression Rating Scale (HDRS) score <12 for the clinical stability criteria.

Help-seeking youth at UHR were recruited from the ETAP within the Department of Psychiatry at Dokuz Eylul University Hospital based on meeting criteria for one of three prodromal syndromes evaluated with the Structured Interview for Psychosis-Risk Syndromes/Scale of Psychosis-Risk Symptoms^35,36^; (1) Brief intermittent psychotic symptom syndrome; (2) Attenuated (subthreshold) positive symptom syndrome; or (3) Genetic risk and deterioration syndrome. In the current study, 43 UHR participants were classified as attenuated positive symptoms syndrome, 13 as brief intermittent psychotic symptom syndrome and 8 as genetic risk and deterioration syndrome. The healthy control subjects were recruited from the local community through advertisements, who had no prior or current history of psychiatric disorders or family history of psychotic disorders.

The participants underwent interviews utilizing the Structured Clinical Interview for DSM-IV Axis I Disorders^37^. Scale for the Assessment of Positive Symptoms (SAPS)^38^ and the Brief Negative Symptom Scale (BNSS)^39–41^ were used to assess current positive and negative symptoms in patients. BNSS is a next-generation negative symptom scale with good internal consistency, convergent and discriminant validity. It has some advantages over widely used scales (i.e., SANS), including distinguishing between consummatory and anticipatory anhedonia. The antipsychotic medication dosage was standardized to the chlorpromazine equivalent^42^. The duration of untreated psychosis (DUP) was also calculated. In addition, the current functioning of the patients was evaluated using the Personal Social Performance (PSP) by calculating a total score based on four subscales of socially useful activities, personal and social relationships, self-care and disturbing and aggressive behaviors^43,44^. A higher total score in the PSP represents better functioning.

The Dokuz Eylul Hospital Ethics Committee for non-interventional studies approved the study protocol, and all participants provided written informed consent. All subjects were native speakers of Turkish. The exclusion criteria for the FEP, UHR and FHP groups and healthy controls were prior medical conditions, ongoing alcohol or substance abuse, or previous neurological disorders. While age and education levels across groups were significantly different (see Table 4 and Table S8), with UHR significantly less educated and younger than NC and FHP, and with FEP significantly less educated than NC. Therefore, years of education and age were added to our regression models.

**Table 4:** Demographic and clinical data of first-episode of psychosis (FEP), ultra-high risk (UHR), family history of psychosis, and neurotypical controls (NC).

	FEP (*n* = 53)	UHR (*n* = 64)	FHP (*n* = 39)	NC (*n* = 34)
	Mean (s.d)	Mean (s.d)	Mean (s.d)	Mean (s.d)
Age	20.91 (4.49)	20.19 (4.03)	22.38 (3.82)	22.33 (4.25)
Sex (m/f)	(27/26)	(31/33)	(16/ 23)	(11/23)
Education	11.29(2.78)	11.62(2.20)	12.64 (2.43)	14.44 (2.51)
BNSS	32.5 (18.1)	22.4 (15.0)	3.90 (7.84)	---
HDRS	4.06 (3.91)	7 (4.85)	3.72 (4.42)	---
Age of onset	21.66 (6.4)	---	---	---
SAPS FTD	3.43 (4.35)	2.05 (3.66)	0.05 (0.33)	---
SAPS Hallucinations	3.63 (4.67)	---	---	---
SAPS Delusions	9.30 (8.03)	---	---	---
SAPS Total	15.9 (12.05)	---	---	---
PSP Total	39.2 (15.3)	51.41 (12.95)	76.9 (15.93)	---
CPZ equivalents	222 (250)	---	---	---
DUP (weeks)	13.9 (22.0)	---	---	---

*SAPS* Scale for the Assessment of Positive Symptoms, *FTD* Formal Thought Disorder, *BNSS* Brief Negative Symptom Scale, *HDRS* Hamilton Depression Rating Scale, *PSP* Personal Social Performance.

### Linguistic testing

All subject interviews took place in the first-episode program of the ETAP at the Department of Psychiatry, Dokuz Eylul University Hospital. Participants were shown eight pictures from the Thematic Apperception Test^45^ in sequence and were asked to talk about each picture for one minute. If participants stopped speaking before the minute was up, they were given a non-directive prompt such as, “Can you say more?” or “What else do you see?”. At the end of the minute, the interviewer asked the participants to clarify any odd or unusual statements they had made. After this, the next picture was shown. The entire process lasted approximately nine minutes. All interviews were recorded on audiotape and later anonymized to remove any identifying information about the participants and their diagnoses. Audio files were transcribed using Whisper 7.4 (https://openai.com/whisper/^46^;. Then, two native Turkish speakers, who were unaware of the participants’ group identifications, checked Whisper’s transcriptions and edited each file. Following the same methodology of ref. ^10^, the statistical analysis below is based on speech generated in response to four randomly selected pictures from the TLI: the first two and the last two pictures (i.e., a farm scene, a man with a pipe and a woman near a small hexagonal table, a woman opening a living room door, and a human figure/man next to a streetlamp post in the dark).

### Annotation and linguistic variables

Following prior studies^9,10,13,47^, an utterance was defined as a grammatically independent unit of discourse that provides new information. This study employed the same annotation manual and procedure as described in ref. ^10^. NP identification and classification were performed manually through hand annotation by trained raters blind to group status, following the scheme in [10]. After manual annotation was completed, pretrained language models (Turkish BERT for semantic similarity and perplexity; Jina-CLIPv2 for image-text alignment) were applied to the annotated transcripts to extract embeddings and compute similarity metrics, not for NP identification or classification.

During manual annotation, after identifying the utterances, all noun phrases (NPs, including pronouns) were initially identified and categorized into lexical NPs (involving a lexical noun or content word as the head of the NP), lexical pronouns, or null (present only through agreement morphology and syntax) (Step 1). Lexical NPs were then further classified into determiner phrases (DPs, headed by a determiner) or “bare” NPs, lacking a determiner (Step 2). DPs were subsequently subclassified as indefinite or definite (Step 3). Indefinite DPs included NPs with indefinite determiners (e.g., *iki* (“two”) + NP, *birkac* (“a few”) + NP, *birçok* (“many“) + NP, and *bir* (“a/one”) + NP), which typically introduce new entities into the discourse. Definite DPs included those used with deictic determiners like *bu/o* (“this”), which refer to a specific individual.

For bare NPs, such as *kadın var* (lit. woman there-is, “There is a woman”), we checked if they were anaphoric to a previously introduced entity (“Bare anaphoric NPs”) or if they referred to a specific entity due to the use of an accusative or other case marker (“Bare specific NPs”)^48,49^ In Turkish, accusative-marked NPs have the case suffix *-(y)I*, with variants *-(y)ı*, *-(y)u*, *-y(ü)* due to vowel harmony (See Table 1). If a bare NP was neither anaphoric nor had a specificity-inducing case marker, it was classified as either a “Bare indefinite NP” or a “Bare generic NP”. In these cases, grammatical context indicators were checked to confirm the non-definite classification (i.e., indefinite or generic). For instance, in *Elinde kitaplar var* (lit. in her hands books there-are, “In her hands there are books”), the plural morpheme on *kitaplar* indicates an indefinite quantification over a set of books. Similarly, in *Köy hayatını anlatan bir şey* (“It is a thing that tells about village life”), the bare NP refers to village life in general^49,50^.

Finally, if the bare NP did not fit any of the previous classifications, it was categorized as “Bare-residual”, such as in *Elinde kitap var* (“There is [a] book in her hand”), where the Turkish NP is bare, without determiners or case, and lacks contextual indicators of indefiniteness or genericity. Our final annotation involved null subjects, which are grammatical subjects identified via agreement morphemes on verbs without being lexically explicit (e.g., *Genç ve güzel* [lit. young and beautiful is]: “She is young and beautiful.”). Turkish, being a “pro-drop” language, allows such null subjects freely (See Table 1 above for the illustration of the annotated NP types.).

We also noted some issues with referential anomalies in the use of these NPs. For instance, in *Dudağında rujları* (which translates to “her lips with lipsticks”), the reference is not clear, and the description appears to be incorrect. Similarly, in *adam tecavüze başlayacak gibi* (“The man looks like he’s about to start raping”), the instance of violence is not evident in the image.

### Reliability analysis

To assess annotation reliability, an independent rater—not involved in data collection, preparation of the annotation manual, or study design—was trained to apply the coding scheme and annotated 77% of the dataset. The proportion of disagreement with the primary rater was 2%, corresponding to 98% observed agreement. To account for chance agreement, we additionally calculated Cohen’s *κ*^51^, which yielded *κ* = 0.96, indicating *almost perfect* agreement according to conventional benchmarks^52^. Given that all disagreements were subsequently resolved by checking criteria, the final dataset used for statistical analyses represented 100% agreement.

### Data analysis

Statistical analysis was performed using R (version 2021.09.1 Build 372). To model the count data—specifically, the frequency of particular linguistic constructions—a Poisson regression was applied using the generalized linear model function (glmer). In this model, Group was treated as a categorical predictor, with the total number of words included as an offset term. This adjustment accounted for variations in the number of words produced by each participant and converted the predicted variable from a count to a rate.

The library sjPlot was used to generate tables with default estimates on the log scale, reflecting group differences as rate differences on this scale. The libraries jtools, ggplot2, and gridExtra were utilized for creating plots. Because there were significant differences in years of education and age across groups (Table S9 for model comparisons between (Model 1) and (Model 2) without education and age as predictors), these variables were included as covariates in each model, after centering. The results presented below are after controlling for years of education and age. However, model comparisons indicated that excluding years of education and age for bare residual NPs, indefinite DP, and null subjects significantly improves the models (see Table S10 for model 1 including education and age for indefinite DP, bare-residuals, and null subjects and Table S11 for model 2 excluding education and age for NP types), and thus these three variables are reported without controlling these two factors. Additionally, instances where certain NP types (e.g., bare NPs) did not appear are represented as 0, and these instances were incorporated into the data analysis. False discovery rate (FDR) was applied to correct *p* values for each group within NP types and is reported as *q* values.t *p* = <0.001.

### Distributions of NP types over narrative time

We analyzed the distributions of the NP distances. For example, if speakers use fewer NPs of a given type overall, those NPs might be evenly spread throughout the narrative, or else clustered in one portion. To assess differences in the dynamic distributions over narrative time, we aggregated all distances for each group and tested whether the distances for four referential expressions (definite DP, indefinite DP, bare-indefinite NP, and bare-residual NP) followed exponential distributions. The reason these four NP types were sub-selected is that, for definites and indefinites, we predict a non-Poisson-like distribution. This involved performing 16 tests in total (4 distributions across 4 groups), with the average distances used as the lambda parameter. We employed Kolmogorov–Smirnov tests, in which each empirical distribution was compared against 100,000 simulated exponential distributions matched in length and λ to the observed data. K-S tests were restricted to four NP types with sufficient occurrences to ensure statistical validity, excluding rare categories such as bare specific and bare generic NPs. The test compared the actual distribution to these 100,000 samples, and the percentage of significant p-values was calculated. The goal of this approach was to determine the likelihood that the distances in each case follow an exponential distribution, suggesting that the referential expressions behaved according to a Poisson process.

### NLP metrics and NP types

Models for semantic similarity (word-to-word and text-to-picture), perplexity, and NP distances are shown in Supplementary Fig. 1.

### Semantic similarity analysis

Semantic similarity has been used to quantify and analyze the coherence of thought and language in individuals experiencing psychotic disorders^23,30,53^. To find semantic similarity, each transcript is first divided into tokens (*t*₁, *t*₂, …, *t*_k_), which are transformed into numerical representations called embeddings (*e*₁, *e*₂, …, *e*_n_) using language models (LMs). Here, *n* represents the total number of tokens. For a processed transcript W = (*w*₁, *w*₂, …, *w*_n_), its overall semantic similarity is calculated as the average cosine similarity between every pair of consecutive embeddings, as in the following:

1 $${semantic}\_{similarity}:=\frac{1}{n-1}{\sum }_{i=1}^{n-1}{\rm{cosine}}\_{\rm{similarity}}({{\rm{e}}}_{{\rm{i}}},{{\rm{e}}}_{{\rm{i}}+1})$$

In our experiments, we applied a monolingual BERT model^26^ for Turkish^27^. The model was pre-trained on 200 GB raw Turkish text which were collected from common crawl corpuses, Twitter, articles, Wikipedia and other sources. First, we removed all punctuations and stop words from the transcripts. Second, we encoded all tokens and convert to their embeddings. Third, we calculated the semantic similarity for each participant as in Eq. (1). It should be noted that we decided to remove stop words and punctuation because we were examining the following three complementary linguistic dimensions:

1. Referential structure (via manually annotated noun phrases, sensitive to grammatical structure),
2. Semantic similarity (lexical-conceptual content derived from embeddings), and
3. Probabilistic predictability (perplexity from contextual probability distributions).

By removing function words and punctuation only for the semantic similarity metric, we sought to ensure that this measure captured conceptual semantic proximity rather than overlap in grammatical scaffolding. Importantly, this preprocessing step did not affect the referential (NP-based) or perplexity analyses, which relied on the full transcriptions.

Nevertheless, to ensure that our main results were not artifacts of this choice, we repeated the analyses, including punctuation and stop words. The direction and significance of group differences remained unchanged, confirming the robustness of our findings (please see Supplementary Fig. 1 for the results with stop words and punctuations).

### Semantic perplexity analysis

In addition to semantic similarity, we analyzed perplexity (PPL) to quantify the predictability of its constituent linguistic units. PPL has been validated as a reliable marker of speech coherence, particularly in detecting cognitive decline through its ability to measure discourse-level coherence patterns^54^. We utilized pseudo-perplexity (PPPL), a modified metric proposed in the study^55^ that uses similar mathematical foundations since language models cannot calculate PPL. For a tokenized sentence W = (*w*₁, *w*₂, …, *w*_*n*_), the probability of each token wᵢ was calculated based on the log-likelihood of the word wᵢ (given the sequence excluding wᵢ). The PPPL was then computed as:

2 $${\text{PPPL}}={\text{exp}}\left(\frac{1}{n}\mathop{\sum }\limits_{i=1}^{n}-{\text{log}}P\left({W}_{i}\mathrm{|context}\right)\right)$$

PPPL scores were calculated for each token in the transcripts using the Turkish BERT model. These scores were then averaged across tokens to derive a single composite PPPL score per participant.

### Multimodal similarity analysis: from image to text

The Jina-CLIPv2 model^56^ is a vision-language framework pre-trained to encode and compare multimodal (image-text) representations for many languages, including Turkish. For each participant, cosine similarity scores were computed between texts and their corresponding image. These pairwise similarity values were aggregated into a mean image-sentence similarity score per participant.

### Relationship among semantic measures and referentiality

We employed Generalized Estimating Equations (GEE) to analyze clustered data, accounting for within-participant correlations. The Gaussian family (identity link) was used for continuous outcomes (e.g., semantic similarity scores). Fixed effects included experimental group (reference: NC), linguistic predictors (e.g., NP types), and covariates (age, gender, education, picture number). An exchangeable correlation structure was assumed, where all observations within a participant share equal correlation. FDR was applied to correct *p* values for each group within all domains (i.e., semantic, perplexity, and multimodal similarity analysis) and is reported as *q* values in this paper. The deviance goodness-of-fit test was applied to assess how well the GEE models fit the observed data.

To understand how linguistic variables correlate with clinical symptoms, we used the Spearman package from the scipy library to conduct partial correlations for the three clinical symptoms (BNSS, SAPS Negatives, and SAPS FTD) that FEP, FHP, and UHR groups all had (see Table S6 for Spearman partial correlation between symptoms and linguistic variables). We controlled for group, age, education, and gender in the analysis. NP types were added to the model, including all definite NPs, all indefinite NPs, the mean distance for definite NPs, the mean distance for indefinite NPs, and NLP metrics such as semantic similarity, perplexity, and image2text. FDR correction was applied to correlations.

## Supplementary information

Supplementary materials

## Data Availability

Transcripts used for this study are available upon reasonable request, in accordance with the stipulations set by the Research Ethics Committee of Dokuz Eylul University. Feature extraction, statistical analyses, and visualization were carried out using Python 3.9.12, and relevant packages. Scripts are available from the corresponding author upon reasonable request and can be accessed in the following link: https://osf.io/9ny2f/overview?view_only=e8404eb4c4f140428999a8cd353b2d90.
