# Supplementary material for: Three dimensions of speech coherence in people with early psychosis and their family members
Source: Schizophrenia (Heidelb). 2025 Dec 17;12(1):2. doi: 10.1038/s41537-025-00703-0 (PMC12775532; doi:10.1038/s41537-025-00703-0)
Supplement: Supplementary file 1 — Supplementary materials [file 41537_2025_703_MOESM1_ESM.pdf]

Supplementary Materials

Table S1. Semantic similarity and perplexity across groups.

**Variables**	*B*	*SE*	*z*	*p*	*q*	Conf. Interval Lower	Conf. Interval Upper
**Sem-sim**							
Intercept	0.67	0.02	28.22	0.00	0.00*	0.63	0.72
FEP	0.01	0.00	3.06	0.01	0.01*	0.01	0.02
UHR	0.01	0.00	2.68	0.02	0.02*	0.00	0.02
FHR	0.01	0.00	1.66	0.21	0.21	-0.00	0.02
pic2	-0.01	0.00	-1.39	0.30	0.30	-0.01	0.00
pic6	-0.00	0.00	-0.77	0.57	0.57	-0.01	0.00
pic8	-0.00	0.00	-0.64	0.57	0.57	-0.01	0.01
Age	-0.00	0.00	-4.31	0.00	0.00*	-0.00	-0.00
Gender	0.00	0.00	0.68	0.57	0.57	-0.00	0.01
Education	0.00	0.00	0.93	0.56	0.56	-0.00	0.00
Word	0.00	0.01	0.34	0.73	0.73	-0.01	0.01
**Img2Txt**							
Intercept	0.22	0.01	24.32	0.00	0.00*	0.20	0.24
FEP	-0.01	0.00	-2.98	0.00	0.01*	-0.01	-0.00
UHR	-0.00	0.00	-1.46	0.14	0.20	-0.01	0.00
FHR	-0.00	0.00	-1.85	0.06	0.12	-0.01	0.00
pic2	0.04	0.00	22.72	0.00	0.00*	0.03	0.04
pic6	0.02	0.00	11.25	0.00	0.00*	0.02	0.02
pic8	0.04	0.00	19.83	0.00	0.00*	0.04	0.04
Age	0.00	0.00	1.29	0.20	0.24	0.00	0.00
Gender	0.00	0.00	-0.46	0.65	0.71	-0.00	0.00
Education	0.00	0.00	-0.29	0.77	0.77	-0.00	0.00
Word	-0.00	0.00	-1.74	0.08	0.13	-0.01	0.00
**Perplexity**							
Intercept	1.66	0.16	10.64	0.00	0.00*	1.35	1.96
FEP	0.01	0.04	0.23	0.82	0.98	-0.07	0.09
UHR	0.03	0.04	0.73	0.47	0.73	-0.05	0.10
FHR	-0.01	0.04	-0.14	0.89	0.98	-0.08	0.07

pic2	-0.10	0.04	-2.68	0.01	0.04*	-0.17	-0.03
pic6	-0.06	0.03	-1.87	0.06	0.23	-0.13	0.00
pic8	-0.05	0.04	-1.32	0.19	0.35	-0.12	0.02
Age	-0.00	0.00	-1.31	0.19	0.35	-0.01	0.00
Gender	0.00	0.03	0.01	0.99	0.99	-0.05	0.05
Education	0.00	0.01	0.20	0.84	0.98	-0.01	0.01
Word	-0.05	0.03	-1.61	0.11	0.30	-0.10	0.01

Table S2: Associations of NP types and NP mean distances with word-to-word and image-to-text semantic similarity, and perplexity Generalized Estimating Equations (GEE) model.

	Sem-sim					Img2Txt					Perplexity				
NP Feature	*B*	*se*	*z*	*p*	*q*	*B*	*SE*	*z*	*p*	*q*	*B*	*se*	*z*	*p*	*q*
Indefinite DP	0.11	0.10	1.06	0.29	0.46	0.01	0.02	0.73	0.47	0.51	0.84	0.34	2.41	0.02	0.06
Definite DP	-0.10	0.04	-2.33	0.02	0.04*	-0.01	0.02	-0.64	0.52	0.58	-0.58	0.38	-1.51	0.13	0.28
Bare anaphoric NP	-0.02	0.06	-0.34	0.74	0.74	0.09	0.02	3.53	0.00	0.00*	-2.48	0.46	-5.30	0.00	0.00*
Bare specific NP	0.35	0.21	1.67	0.09	0.18	-0.06	0.14	-0.45	0.65	0.72	-0.71	2.16	-0.33	0.74	0.92
Bare indefinite NP	0.08	0.05	1.49	0.00	0.25	-0.02	0.02	-1.02	0.31	0.38	1.37	0.55	2.48	0.01	0.07
Bare generic NP	-0.14	0.08	-1.80	0.07	0.16	0.01	0.04	0.33	0.74	0.82	-3.96	0.69	-5.68	0.00	0.00*
Bare-residual	0.10	0.04	2.35	0.02	0.04*	0.00	0.01	-0.48	0.63	0.69	-0.35	0.17	-2.03	0.04	0.14
Null subject	-0.15	0.03	-4.48	0.00	0.00*	0.09	0.01	5.90	0.00	0.00*	-0.83	0.29	-2.79	0.00	0.03*
Lexical pronoun	0.07	0.07	1.14	0.25	0.40	-0.04	0.03	-1.47	0.14	0.22	0.74	0.65	1.13	0.26	0.43
All definite NPs	-0.09	0.02	-4.13	0.02	0.00*	0.05	0.01	4.31	0.00	0.00*	-0.81	0.20	-3.96	0.00	0.00*
All indefinite NPs	0.09	0.03	2.44	0.02	0.03*	-0.01	0.01	-0.82	0.41	0.50	-0.05	0.18	-0.33	0.75	0.93
Referential Anom[1]	0.03	0.06	0.07	0.94	0.94	-0.10	0.06	-1.73	0.08	0.15	3.11	1.42	2.20	0.03	0.03*
Definite DP MD[2]	0.01	0.01	0.77	0.44	0.50	0.01	0.01	0.21	0.82	0.82	0.01	0.01	-0.39	0.69	0.69
Indefinite DP MD	-0.01	0.01	-2.21	0.027	0.07	-0.01	0.01	-1.42	0.15	0.38	0.01	0.01	-1.34	0.17	0.35

Bare-residual MD	-0.01	0.01	-1.67	0.094	0.15	-0.01	0.01	-0.85	0.39	0.62	0.01	0.01	-0.18	0.85	0.85
Bare indefinite NP MD	-0.01	0.01	-0.96	0.33	0.44						0.01	0.01	0.51	0.60	0.51

Note: We reported the coefficient (*B*), standard error (*se*), z score (*z*), p values before correction (*p*), and p values after correction (*q*) for every group, to make the table concise, with the complete table of results available in supplementary materials. [1]Anom corresponds to anomalies. [2]MD: mean distance.

Table S3. Relationship between semantic similarity and referentiality variables.

Group	NP types	*B*	*SE*	z	*p*	*q*
FEP	Indefinite DP	0.013	0.004	3.071	0.002	0.008*
	Definite DP	0.011	0.004	2.713	0.006	0.021*
	Bare anaphoric NP	0.013	0.004	3.009	0.002	0.010*
	Bare specific NP	0.013	0.004	3.122	0.002	0.007*
	Bare indefinite NP	0.013	0.004	3.084	0.002	0.007*
	Bare generic NP	0.013	0.004	3.129	0.002	0.006*
	Bare-residual	0.011	0.004	2.847	0.004	0.016*
	Null subject	0.012	0.004	3.056	0.002	0.006*
	Lexical pronoun	0.013	0.004	3.059	0.002	0.008*
	All definite NPs	0.011	0.004	2.690	0.007	0.020*
	All indefinite NPs	0.011	0.004	2.807	0.005	0.018*
	Referential anomalies	0.013	0.004	3.010	0.003	0.010*
	Definite DP mean distance	0.013	0.006	2.324	0.020	0.054
	Indefinite DP mean distance	0.012	0.006	2.111	0.035	0.070
	Bare indefinite NP mean distance	0.014	0.006	2.456	0.014	0.037
	Bare-residual mean distance	0.012	0.006	2.016	0.044	0.088
FHP	Indefinite DP	0.007	0.004	1.634	0.095	0.225
	Definite DP	0.006	0.004	1.466	0.135	0.224
	Bare anaphoric NP	0.007	0.004	1.606	0.102	0.238
	Bare specific NP	0.007	0.004	1.635	0.096	0.177
	Bare indefinite NP	0.007	0.004	1.639	0.095	0.223
	Bare generic NP	0.007	0.004	1.613	0.100	0.168
	Bare-residual	0.006	0.004	1.565	0.110	0.216
	Null subject	0.008	0.004	2.094	0.034	0.066
	Lexical pronoun	0.007	0.004	1.729	0.078	0.185
	All definite NPs	0.006	0.004	1.516	0.123	0.237
	All indefinite NPs	0.006	0.004	1.586	0.106	0.207
	Referential anomalies	0.008	0.004	1.907	0.057	0.124
	Definite DP mean distance	0.011	0.006	1.865	0.062	0.054
	Indefinite DP mean distance	0.011	0.006	1.669	0.095	0.152
	Bare indefinite NP mean distance	0.009	0.006	1.542	0.123	0.037
	Bare-residual mean distance	0.007	0.006	1.131	0.258	0.088
UHR	Indefinite DP	0.012	0.004	2.747	0.006	0.017*
	Definite DP	0.011	0.004	2.668	0.008	0.021*
	Bare anaphoric NP	0.012	0.004	2.703	0.007	0.019*
	Bare specific NP	0.012	0.004	2.692	0.007	0.020*
	Bare indefinite NP	0.012	0.004	2.737	0.006	0.017*
	Bare generic NP	0.012	0.004	2.762	0.006	0.016*
	Bare-residual	0.012	0.004	2.747	0.006	0.017*
	Null subject	0.012	0.004	2.876	0.004	0.009*
	Lexical pronoun	0.012	0.004	2.812	0.005	0.014*

All definite NPs	0.011	0.004	2.551	0.011	0.024*
All indefinite NPs	0.011	0.004	2.727	0.007	0.018*
Referential anomalies	0.012	0.005	2.623	0.009	0.024*
Definite DP mean distance	0.012	0.005	2.131	0.033	0.504
Indefinite DP mean distance	0.006	0.006	1.080	0. 280	0.152
Bare indefinite NP mean distance	0.011	0.005	2.033	0.042	0.197
Bare-residual mean distance	0.011	0.006	2.014	0.044	0.344

Table S4. Relation between image to text scores and referentiality variables.

Group	NP types	*B*	*SE*	*z*	*p*	*q*
FEP	Indefinite DP	-0.0058	0.002	-2.712	0.007	0.015*
	Definite DP	-0.0059	0.002	-2.737	0.006	0.014*
	Bare anaphoric NP	-0.0052	0.002	-2.481	0.013	0.024*
	Bare specific NP	-0.0058	0.002	-2.697	0.007	0.015*
	Bare indefinite NP	-0.0057	0.002	-2.685	0.007	0.016*
	Bare generic NP	-0.0058	0.002	-2.685	0.007	0.016*
	Bare-residual	-0.0057	0.002	-2.639	0.008	0.018*
	Null subject	-0.0055	0.002	-2.602	0.009	0.017*
	Lexical pronoun	-0.0057	0.002	-2.716	0.007	0.015*
	All definite NPs	-0.0048	0.002	-2.219	0.027	0.049*
	All indefinite NPs	-0.0056	0.002	-2.612	0.009	0.020*
	Referential anomalies	-0.0049	0.002	-2.299	0.022	0.010*
	Definite DP mean distance	-0.0052	0.003	-1.926	0.054	0.697
	Indefinite DP mean distance	-0.0067	0.003	-2.093	0.036	0.145
	Bare indefinite NP mean distance	-0.0053	0.003	-1.923	0.055	0.218
	Bare-residual mean distance	-0.0054	0.003	-1.904	0.057	0.228
FHP	Indefinite DP	-0.0037	0.002	-1.647	0.100	0.182
	Definite DP	-0.0037	0.002	-1.663	0.096	0.177
	Bare anaphoric NP	-0.0032	0.002	-1.439	0.150	0.236
	Bare specific NP	-0.0037	0.002	-1.644	0.100	0.184
	Bare indefinite NP	-0.0037	0.002	-1.640	0.101	0.185
	Bare generic NP	-0.0037	0.002	-1.636	0.102	0.187
	Bare-residual	-0.0037	0.002	-1.636	0.102	0.187
	Null subject	-0.0045	0.002	-1.977	0.048	0.076
	Lexical pronoun	-0.0039	0.002	-1.770	0.077	0.141
	All definite NPs	-0.0033	0.002	-1.465	0.143	0.219
	All indefinite NPs	-0.0036	0.002	-1.635	0.102	0.187
	Referential anomalies	-0.0033	0.002	-1.486	0.137	0.124
	Definite DP mean distance	-0.0046	0.003	-1.571	0.116	0.310
	Indefinite DP mean distance	-0.0056	0.003	-1.619	0.105	0.281
	Bare indefinite NP mean distance	-0.0037	0.003	-1.263	0.207	0.551
	Bare-residual mean distance	-0.0035	0.003	-1.156	0.248	0.495
UHR	Indefinite DP	-0.0026	0.002	-1.170	0.242	0.333
	Definite DP	-0.0027	0.002	-1.201	0.230	0.333
	Bare anaphoric NP	-0.0023	0.002	-1.048	0.295	0.360
	Bare specific NP	-0.0026	0.002	-1.171	0.241	0.332
	Bare indefinite NP	-0.0026	0.002	-1.186	0.236	0.357
	Bare generic NP	-0.0027	0.002	-1.193	0.233	0.320
	Bare-residual	-0.0064	0.013	-0.484	0.235	0.347
	Null subject	-0.0027	0.002	-1.217	0.223	0.273
	Lexical pronoun	-0.0029	0.002	-1.315	0.189	0.256
	All definite NPs	-0.0021	0.002	-0.965	0.335	0.349

All indefinite NPs	-0.0026	0.002	-1.179	0.238	0.368
Referential anomalies	-0.0022	0.002	-0.995	0.320	0.024*
Definite DP mean distance	-0.0024	0.003	-0.876	0.381	0.697
Indefinite DP mean distance	-0.0028	0.003	-0.859	0.390	0.624
Bare indefinite NP mean distance	-0.0021	0.003	-0.771	0.440	0.705
Bare-residual mean distance	-0.0024	0.003	-0.878	0.380	0.608

Table S5. Results of quasi-deviance goodness-of-fit tests for GEE models.

Predictor	Deviance (Full Model)	Deviance (Null Model)	Chi-Squared	*p*
Indefinite DPs	1.40	1.46	34.12	0.0003
Definite DPs	1.40	1.46	32.37	0.0007
Anaphoric	1.41	1.46	28.21	0.0030
Specific due to case-marking	1.41	1.46	28.72	0.0025
Indefinite	1.40	1.46	30.24	0.0015
Generic	1.41	1.46	29.06	0.0022
Bare-residual	1.38	1.46	41.73	<0.0001
Null subject	1.38	1.46	45.66	<0.0001
Lexical pronoun	1.41	1.46	29.58	0.0018
All definite NPs	1.39	1.46	40.42	<0.0001
All indefinite NPs	1.38	1.46	42.61	<0.0001

Table S6. Relation between perplexity scores and referentiality

Group	NP types	*B*	*SE*	z	*p*	*q*
FEP	Indefinite DP	0.0265	0.041	0.647	0.517	0.712
	Definite DP	0.0191	0.042	0.458	0.647	0.889
	Bare anaphoric NP	0.0134	0.039	0.345	0.730	0.892
	Bare specific NP	0.0270	0.041	0.663	0.507	0.797
	Bare indefinite NP	0.0255	0.041	0.628	0.530	0.729
	Bare generic NP	0.0323	0.041	0.784	0.433	0.596
	Bare-residual	0.0311	0.041	0.762	0.446	0.613
	Null subject	0.0253	0.040	0.635	0.526	0.723
	Lexical pronoun	0.0265	0.041	0.650	0.516	0.709
	All definite NPs	0.0098	0.040	0.246	0.806	0.886
	All indefinite NPs	0.0280	0.041	0.687	0.492	0.773
	Referential anomalies	0.0023	0.042	0.053	0.957	0.957
	Definite DP mean distance	0.0179	0.011	1.699	0.089	0.829
	Indefinite DP mean distance	0.0145	0.013	1.143	0.253	0.388
	Bare indefinite NP mean distance	0.016	0.011	1.521	0.128	0.135
	Bare-residual mean distance	-0.001	0.001	-1.453	0.151	0.369
FHP	Indefinite DP	0.0045	0.040	0.112	0.911	0.960
	Definite DP	0.0014	0.040	0.035	0.972	0.972
	Bare anaphoric NP	-0.008	0.038	-0.216	0.829	0.904
	Bare specific NP	0.0048	0.040	0.120	0.905	0.923
	Bare indefinite NP	0.0039	0.040	0.098	0.922	0.922
	Bare generic NP	0.0031	0.040	0.076	0.939	0.939
	Bare-residual	0.0056	0.040	0.140	0.888	0.955
	Null subject	0.0124	0.038	0.324	0.746	0.871
	Lexical pronoun	0.0085	0.040	0.213	0.832	0.885
	All definite NPs	-0.001	0.038	-0.027	0.978	0.978
	All indefinite NPs	0.0050	0.040	0.125	0.900	0.931
	Referential anomalies	-0.006	0.040	-0.160	0.873	0.873
	Definite DP mean distance	0.0078	0.011	0.691	0.490	0.357
	Indefinite DP mean distance	0.0029	0.014	0.215	0.830	0.405
	Bare indefinite NP mean distance	0.0108	0.011	0.955	0.340	0.256
	Bare-residual mean distance	0.0066	0.012	0.557	0.578	0.369
UHR	Indefinite DP	0.0408	0.039	1.060	0.289	0.455
	Definite DP	0.0360	0.038	0.946	0.344	0.541
	Bare anaphoric NP	0.0289	0.036	0.793	0.428	0.723
	Bare specific NP	0.0380	0.038	0.993	0.321	0.588
	Bare indefinite NP	0.0360	0.038	0.950	0.342	0.627
	Bare generic NP	0.0422	0.039	1.081	0.280	0.440
	Bare-residual	0.0381	0.038	0.998	0.318	0.500
	Null subject	0.0381	0.038	0.998	0.304	0.500
	Lexical pronoun	0.0418	0.038	1.092	0.275	0.432
	All definite NPs	0.0287	0.036	0.790	0.430	0.675

All indefinite NPs	0.0378	0.038	0.992	0.321	0.589
Referential anomalies	0.0239	0.039	0.620	0.536	0.536
Definite DP mean distance	0.0140	0.010	1.338	0.181	0.482
Indefinite DP mean distance	0.0165	0.013	1.298	0.194	0.388
Bare indefinite NP mean distance	0.0178	0.011	1.688	0.091	0.244
Bare-residual mean distance	0.0158	0.011	1.444	0.149	0.369

Table S7. Spearman partial correlation between clinical symptoms and linguistic variables.

Symptoms	Linguistic variables	*rho*	*p*	*q*
BNNS	All definite NPs	-0.14	0.69	0.43
	All indefinite NPs	0.20	0.13	0.23
	All definite NP mean distance	0.04	0.57	0.85
	All indefinite NP mean distance	-0.08	0.32	0.78
	Semantic similarity	0.05	0.54	0.85
	Image to text	-0.02	0.12	0.62
	Perplexity	0.08	0.29	0.78
SAPS FTD	All definite NPs	-0.13	0.12	0.61
	All indefinite NPs	0.03	0.68	0.99
	All definite NP mean distance	0.10	0.26	0.62
	All indefinite NP mean distance	-0.04	0.64	0.99
	Semantic similarity	-0.07	0.39	0.86
	Image to text	-0.05	0.54	0.93
	Perplexity	-0.00	0.99	0.99

Abbreviations: SAPS = Scale for the Assessment of Positive Symptoms; SAPS FTD=Formal Thought Disorder; BNSS = Brief Negative Symptom Scale

Table S8. T-test comparisons of age and education across groups.

Comparisons	Age		Education	
	t (df)	*p*	*t(df)*	*p*
NC vs. FEP	1.341 (68)	>.05	3.304 (81)	.001
NC vs. UHR	2.146 (64)	.035	5.362 (60)	.001
NC vs. FHP	-0.064 (63)	>.05	3.064 (65)	.003
FEP vs. UHR	0.843 (103)	>.05	1.172 (85)	>.05
FEP vs. FHP	-1.541 (82)	>.05	-0.546 (86)	>.05
FHP vs. UHR	2.448 (79)	.016	2.026 (70)	.046

Table S9. Model comparisons between (Model 1) and (Model 2) without education and age as a predictor.

	**Model 1** [a,b]				**Model 2**			
Predictors	Resid Df	Resid. Dev	(Df) Deviance	Pr(>Chi)	Resid Df	Resid. Dev	(Df) Deviance	Pr(>Chi)
Indefinite DP	184	293.62	---	---	186	307.09	(-2) -13.472	.001*
Definite DP	184	398.93	---	---	186	400.89	(-2) -1/9645	.374
Bare anaphoric NP	184	461.97	---	---	186	466.94	(-2) -4.973	.083
Bare specific NP	184	71.745			186	73.223	(-2) -1.477	.477
Bare indefinite NP	184	277.02	---	---	186	279.00	(-2) -1.980	.371
Bare generic NP	184	286.00	---	---	186	286.08	(-2) -0.085	.9581
Bare-residual	184	561.38	---	---	186	579.43	(-2) -18.047	.001*
Null subject	184	416.88	---	----	186	422.93	(-2) -6.0476	.048*
Lexical pronoun	184	397.14	---	---	186	397.87	(-2) -0.723	.696
Referential anomalies	184	300.01	---	---	186	301.54	(-2) -1.5258	.466

Note: Neurotypical controls (NC) are taken as a reference. FEP, UHR and FHP were compared with NC (Neurotypical control)

Table S10. Model 1 including education and age for indefinite DP, bare-residuals, and null subjects

	Indefinite DP	Bare residuals	Null subjects
Predictors	Log-Mean<br>P	Log-Mean<br>P	Log-Mean<br>P
(Intercept)	-2.91<br>(-3.12 – -2.70)<br>**<0.001**	-2.77<br>(-3.07 – -2.48)<br>**<0.001**	-1.00<br>(-1.22 – -0.79)<br>**<0.001**
FEP	-0.03<br>(-0.13 – 0.07)<br>0.535	0.25<br>(0.11 – 0.40)<br>**0.001**	-0.24<br>(-0.34 – -0.13)<br>**<0.001**
FHP	-0.03<br>(-0.13 – 0.08)<br>0.616	0.10<br>(-0.06 – 0.25)<br>0.238	0.00<br>(-0.10 – 0.11)<br>0.968
UHR	-0.06<br>(-0.15 – 0.04)<br>0.251	0.01<br>(-0.14 – 0.16)<br>0.941	-0.09<br>(-0.19 – 0.01)<br>0.066
education	0.02<br>(0.00 – 0.03)<br>**0.012**	-0.03<br>(-0.05 – -0.00)<br>**0.018**	0.02<br>(0.01 – 0.04)<br>**0.002**
age	0.00<br>(-0.01 – 0.01)<br>0.544	-0.01<br>(-0.03 – 0.00)<br>0.106	-0.01<br>(-0.02 – 0.00)<br>0.131
Observations	190	190	190
R^2 Nagelkerke	0.114	0.189	.212

Note: Neurotypical controls (NC) are taken as a reference. FEP, UHR and FHP were compared with NC (Neurotypical control

Table S11. Model 2 excluding education and age for NP types. Incidence rates (log scale) of different NP types in the groups relative to neurotypical controls (reference). First-episode Psychosis (FEP), Ultra-High Risk (UHR), Family History of Psychosis and Neurotypical Control (NC).

	Indefinite DP	Definite DP	Bare anaphoric NP	Bare specific NP	Bare indefinite NP	Bare generic NP	Bare residual NP	Null subject	Lexical Pronoun	Ref. Anomalies
Predictors	*Log-Mean* (*p*)	*Log-Mean* (*p*)	*Log-Mean* (*p*)	*Log-Mean* (*p*)	*Log-Mean* (*p*)	*Log-Mean* (*p*)	*Log-Mean* (*p*)	*Log-Mean* (*p*)	*Log-Mean* (*p*)	*Log-Mean* (*p*)
Intercept	-2.57 (-2.64 – -2.50) **<0.001**	-3.04 (-3.13 - 2.95) **<0.001**	-3.59 (-3.70 -3.47) **<0.001**	-8.58 (-9.96 – -7.19) **<0.001**	-3.23 (-3.33 – -3.14) **<0.001**	-6.27 (-6.75 – -5.87) **<0.001**	-3.43 (-3.54 – -3.32) **<0.001**	-0.82 (-0.89 – -0.75) **<0.001**	-3.62 (-3.74 -3.51) **<0.001**	-5.84 (-6.21 – -5.50) **<0.001**
FEP	-0.06 (0.15 – 0.04) 0.251	-0.38 (-0.52 -0.25) **<0.001**	-0.23 (-0.40 -0.06) **0.007**	-16.98 (-3991.79 – 3957.84) 0.993	-0.00 (-0.13 – 0.13) 0.990	0.57 (0.03 – 1.12) 0.043	0.30 (0.16 – 0.44) **<0.001**	-0.28 (-0.38 – -0.17) **<0.001**	0.11 (0.05 - 0.27) 0.182	1.38 (1.00 – 1.79) **<0.001**
FHP	-0.05 (-0.15 – 0.05) 0.323	-0.13 (-0.27 – 0.00) 0.054	-0.20 (-0.37 -0.02) **0.026**	-0.62 (-3.02 – 1.78) 0.624	0.03 (-0.11 – 0.16) 0.713	0.36 (1.08 – 0.33) 0.318	0.12 (0.03 – 0.28) 0.116	-0.05 (-0.15 – 0.06) 0.363	-0.11 (0.28 – 0.06) 0.210	0.68 (0.25 – 1.13) **0.003**
UHR	-0.10 (-0.20 – -0.01) **0.028**	-0.03 (-0.15 – 0.09) 0.650	-0.09 (0.24 – 0.06) 0.240	1.54 (0.06 – 3.03) **0.042**	0.01 (-0.13 – 0.12) 0.901	0.17 (-0.37 – 0.75) 0.540	0.08 (-0.06 – 0.23) 0.265	-0.10 (-0.20 -0.01) **0.038**	-0.22 (-0.38 -0.06) **0.008**	0.99 (0.61 – 1.41) **<0.001**
Observation	190	190	190	190	190	190	190	190	190	190
R^2 Nagelkerke	0.031	0.200	0.050	0.253	0.002	0.065	0.104	0.178	0.118	0.335

Note: Neurotypical controls (NC) are taken as a reference. FEP, UHR and FHP were compared with NC (Neurotypical control)

(A) Semantic Similarity

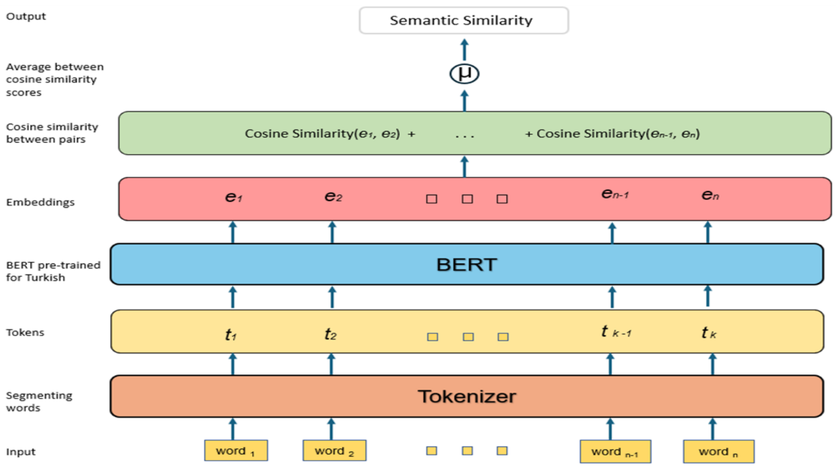

(B) Perplexity

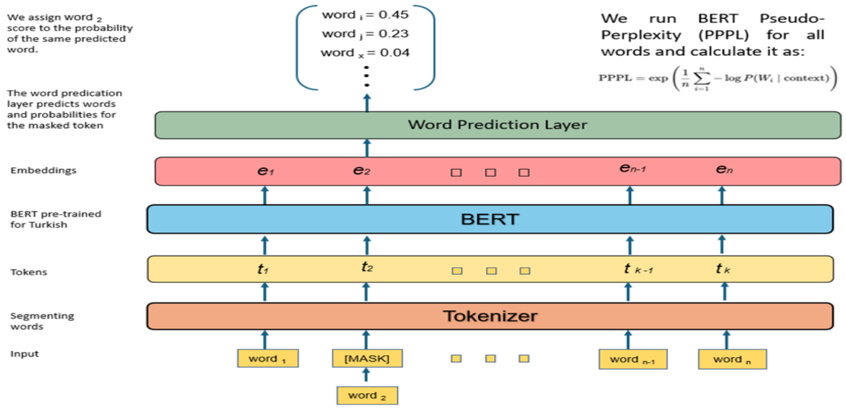

(C) Semantic Similarity between image and text using Jina-CLIPv2

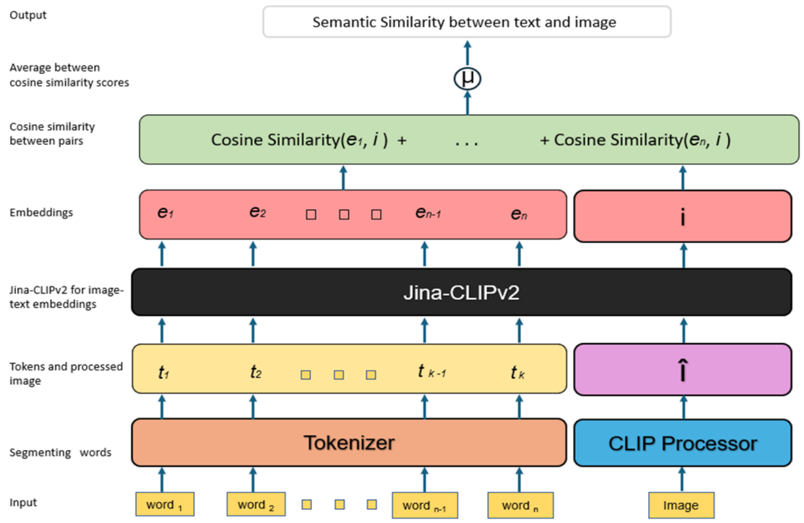

(D) Noun phrase (NP) distance. We show an example of how to compute *indefinite* DP distance:

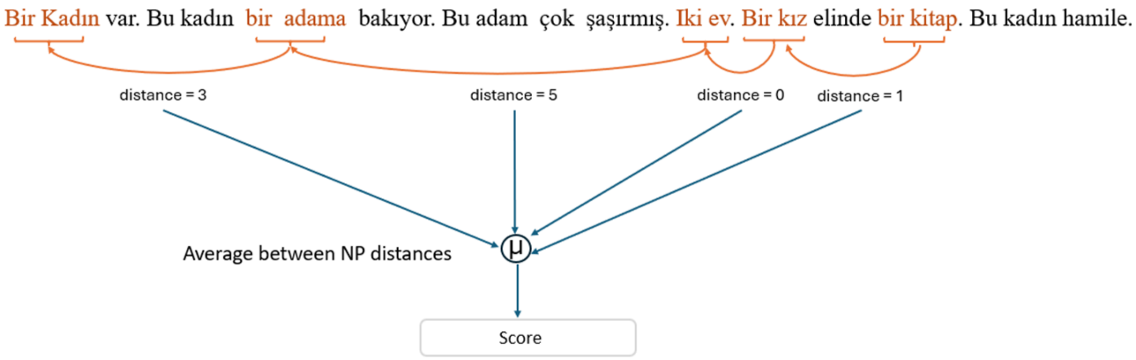

Figure 1. Models for semantic similarity (word-to-word and text-to-picture), perplexity, and NP distance.

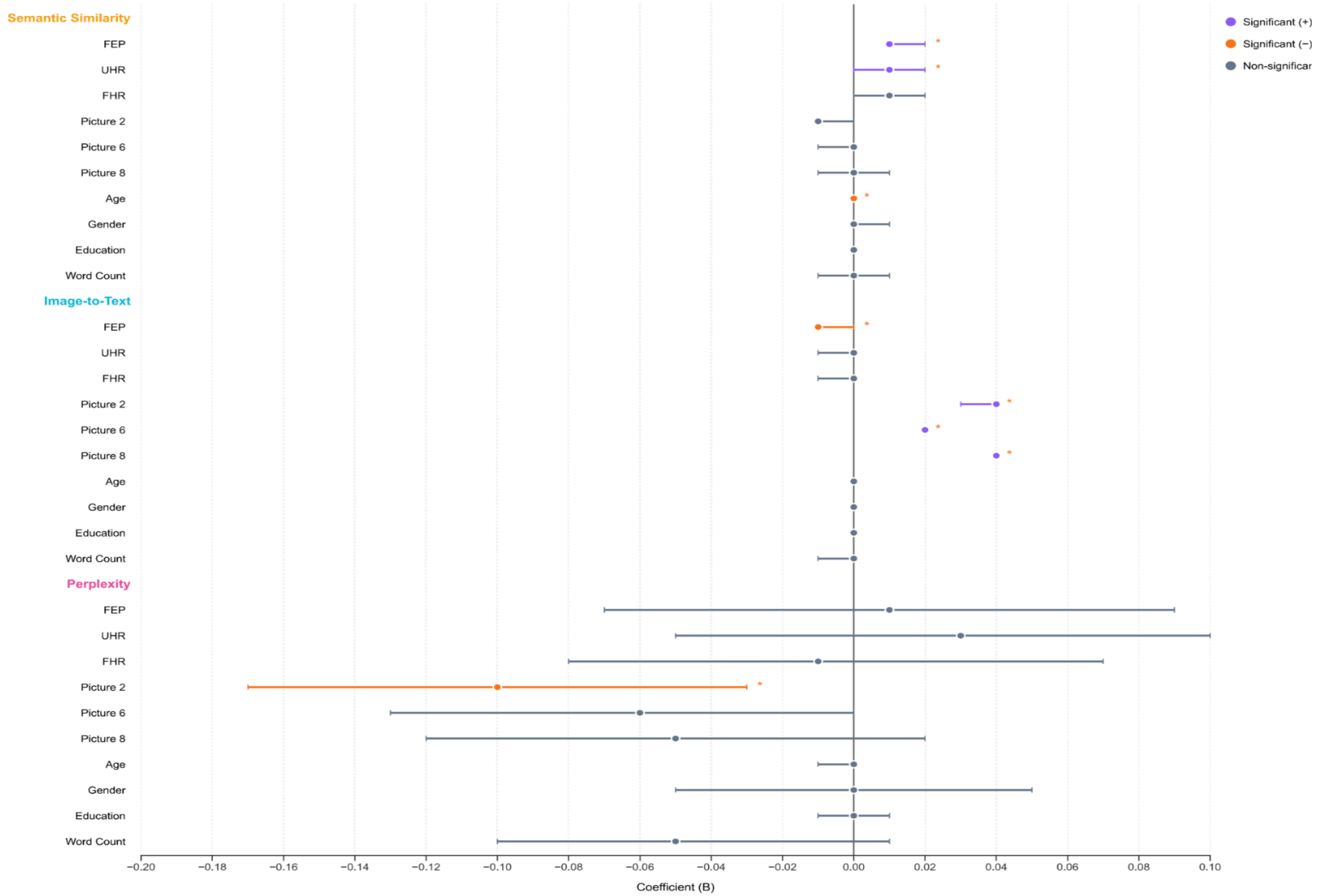

Figure 2. BERT with stop words and punctuations. Forest plot showing regression coefficients with 95% confidence intervals for group effects (FEP, UHR, FHR) and co-variants on three outcome measures. Purple indicates significant positive effects, orange indicates significant negative effects, and grey indicates non-significant effects ($q < 0.05$ after correction). First-episode Psychosis (FEP), Ultra-High Risk (UHR), and Family History of Psychosis (FHP).
